# Commentary: A Longitudinal Randomized Controlled Trial Protocol to Evaluate the Effects of Wuqinxi on Dynamic Functional Connectivity in Parkinson's Disease Patients

**DOI:** 10.3389/fnhum.2022.805331

**Published:** 2022-04-01

**Authors:** Yifan Gong, Dongli Wang, Zhibin Liu

**Affiliations:** ^1^The First Clinical Medical School, Henan University of Chinese Medicine, Zhengzhou, China; ^2^Department of AIDS Treatment and Research Center, The First Affiliated Hospital of Henan University of Chinese Medicine, Zhengzhou, China

**Keywords:** Parkinson's Disease, Wuqinxi exercise, traditional Chinese exercise, Protocol, Randomized Controlled (clinical) Trial

Parkinson's disease (PD) is a complex, age-related, and neurodegenerative disease associated with dopamine deficiency (Simon et al., 2020). This condition is characterized by rest tremor, bradykinesia, rigidity and postural instability, and other motor and non-motor symptoms (Jankovic and Tan, 2020). The prevalence of PD ranges from 41 to 1,903 cases per 100,000 (Evans et al., 2020), and about 10% of patients suffer from advanced PD (Muangpaisan et al., 2011). While PD is still incurable, exercise therapy may benefit patients diagnosed with it. For instance, Bayesian network meta-analysis showed tango as an optimal and effective option for improving functional mobility of patients with PD (Tang et al., 2019). Recently, we have carefully read the article with great interest by Li et al. (2021) entitled “A Longitudinal Randomized Controlled Trial Protocol to Evaluate the Effects of Wuqinxi (WQX) on Dynamic Functional Connectivity in Parkinson's Disease Patients” in 2021. After carefully reading their research program, we believe that the following improvements may be more helpful to achieve the real results.

Firstly, if possible, randomizing patients with PD into three groups might be a better study method. Aside from the two groups described by the author, the third group can be defined as those who are waiting for treatment without any additional exercise intervention, and then, giving them WQX or balance training after 24 weeks of follow-up if they are willing to accept. The role of the waiting for the treatment group is more significant and meaningful than that of the healthy control group, which is more conducive to the comparative study of intervention efficacy and disease pathological changes.

Secondly, we suggest that the authors should more clearly describe or define the patients with specific diseases that need to be excluded. For example, patients without or only mild Knee osteoarthritis (KOA) can participate in the study, but severe patients are deemed not suitable for WQX exercise. After all, some exercises in WQX exercise are too intense, and there is a risk of fall-down for older people at the beginning of exercise. Furthermore, we don't think that the patients with an H&Y score of 3 can complete the WQX exercise.

Thirdly, the WQX exercise was created by Hua Tuo in the Han Dynasty, with a set of medical qigong to prevent, cure, and prolong life by imitating the movements of five animals, which are tiger, deer, bear, ape, and crane (Yang and Wu, 2011). The WQX has a strong cultural characteristic of Chinese medicine, and the movements and postures of the five animals are considered to have the effect of balancing the Qi and the blood of the five zang-fu organs according to the theory of Chinese medicine. The WQX is not only a simple imitation of the main movement of these five animals, but it also shows five related functions according to Chinese medicine theory based on a holistic view of health care. The following results are claimed to be achieved when carrying out WQX in relation to the five animals: tiger-xi can relax the liver, regulate the Qi, relax muscles, and activate collaterals; deer-xi can replenish the Qi and kidney, as well as strengthen the waist and stomach; bear-xi can regulate the spleen and stomach, as well as enrich the limbs; ape-xi can nourish the heart and brain, as well as enlighten and benefit wisdom; bird-xi can replenish the lung, broaden the chest, and smoothen the Qi machine. During the exercising of WQX, the cooperation between action and breath is the very key, rather than being a simple action imitation. Thus, the initial WQX exercise needs to be carried out under the guidance of the professionals, and the cognition of patients necessitates being normal or close to the normal level. In this study, ensuring that every participant will really receive the real WQX exercise rather than showing the imitation of movements will directly impact the research results, especially for patients with PD, along with cognitive impairment (i.e., 20 ≤ Montreal Cognitive Assessment (MoCA) score ≤ 25). This condition will be a huge challenge for the research. We advise the researchers to establish a set of higher quality controlling methods for WQX exercise for these special volunteers.

Shortly, we hope our suggestions will be valuable to the study of Li et al. or others in the future. We are expecting that the research carried out by Li et al. will be successful to test their hypothesis as soon as possible, and we are looking forward to getting evidence that WQX exercise can improve clinical symptoms, as well as induce functional and structural changes in the nervous system of patients with PD. We also look forward to seeing WQX as a choice of clinicians and nurses in their practice to serve patients with PD.

## Author Contributions

ZL: designed, analyzed, and wrote the article. YG and DW: collected the data. All authors critically reviewed and proved the final version to be published.

## Funding

This study was supported by the National Special Science and Technology Program on Major Infectious Diseases (Grant Nos. 2017ZX10205502002 and 2017ZX10205502003) and Henan Province TCM Research Project (Grant No. 20-21ZYZD03). The findings and conclusions of this article are only those of the authors and do not represent the views of the funding sources.

## Conflict of Interest

The authors declare that the research was conducted in the absence of any commercial or financial relationships that could be construed as a potential conflict of interest.

## Publisher's Note

All claims expressed in this article are solely those of the authors and do not necessarily represent those of their affiliated organizations, or those of the publisher, the editors and the reviewers. Any product that may be evaluated in this article, or claim that may be made by its manufacturer, is not guaranteed or endorsed by the publisher.
